# Insights Into GLP-1 and GIP Actions Emerging From Vildagliptin Mechanism Studies in Man

**DOI:** 10.3389/fendo.2019.00780

**Published:** 2019-11-08

**Authors:** James E. Foley

**Affiliations:** Retired, Sparta, NJ, United States

**Keywords:** β-cell, α-cell, hypoglycemia, lipo-toxicity, weight, type 2 diabetes

## Abstract

Vildagliptin blocks glucagon like peptide-1 (GLP-1) and glucose-dependent insulinotropic polypeptide (GIP) inactivation of the meal induced increases in GLP-1 and GIP so that elevated GLP-1 and GIP levels are maintained over 24 h. The primary insulin secretion effect of vildagliptin is to improve the impaired sensitivity of the β-cells to glucose in subjects with impaired fasting glucose (IFG) and impaired glucose tolerance (IGT) and in patients with type 2 diabetes mellitus (T2DM); this effect was seen acutely and maintained over at least 2 years in patients with T2DM. Vildagliptin was also associated with improved β-cell function that is likely secondary to the improved metabolic state. Although there was no evidence of restoration of β-cell mass, the preponderance of the vildagliptin data does indicate that for at least 2 years β-cell function was maintained in vildagliptin treated patients but not in the untreated patients. Vildagliptin suppressed an inappropriate glucagon response to an oral glucose challenge in patients with T2DM, to a mixed meal challenge in patients with T2DM and type 1 diabetes mellitus, and to a mixed meal challenge in subjects with IGT and IFG. The improved glucagon response was maintained for at least 2 years in patients with T2DM and there was no change in the glucagon response in normoglycemic individuals. Vildagliptin lowered glucose levels into the normal range without increasing hypoglycemia. These hypoglycemic benefits appear to be secondary in large part to the improved sensitivity of both the β and α-cell to glucose. In the case of the α-cell, if glucose levels are high, GLP-1 attenuates the glucagon levels and if glucose levels are low, GIP increases glucagon levels. Vildagliptin reduces fatty acid flux from the adipocyte leading to reduced liver fat which in turn leads to increased glucose utilization. The reduced glycosuria and reduced lipo-toxicity associated with vildagliptin therapy does not lead to weight gain presumably due to increased fat mobilization and oxidation during meals and to reduced fat extraction from the gut.

## Introduction

The loss of sensitivity of the islets to glucose characterizes type 2 diabetes mellitus (T2DM). Insulin levels are higher than normal, but inadequate to overcome insulin resistance due initially to inappropriate glucagon secretion and to the lipo-toxicity that is characterized by greater triacylglycerol storage in non-fat tissues. Increasing hyperglycemia in T2DM is associated with increasing glucose toxicity and diminished maximum capacity of the β-cells to secrete insulin. The loss of sensitivity of the islets to glucose not only leads to hyperglycemia, but also to increased hypoglycemia (1). The Sandoz (which became Novartis in 1997) interest in glucagon like peptide-1 (GLP-1) followed directly from this problem statement.

At the 1990 EASD it was first reported that GLP-1 was a useful tool in the treatment of T2DM in humans and in 1992 the data was published (2). Since that first report at the EASD Sandoz then Novartis has played a pioneering role in GLP-1 based therapies (3). In 1993 after an aggressive but failed attempt over 2 years to make a non-peptide GLP-1 peptide mimetic, attention switched to inactivating dipeptidyl peptidase-4 (DPP-4) (3) when it was reported that GLP-1 was inactivated solely by DPP-4 (4). The same is true for the inactivation of the other incretin hormone glucose-dependent insulinotropic polypeptide (GIP), but this was not a focus at that time (3). The DPP-4 program was accelerated in 1995 after it was reported that DPP-4 inhibition raised GLP-1 *in vitro* (3, 5). By the end of 1995 using valine pyrrolidide, a known orally active DPP-4 inhibitor from the Sandoz library, it was shown that a DPP-4 inhibitor could lower blood glucose levels in rodents and non-human primates (3). In 1996, using combinatorial chemistry and valine pyrrolidide as a starting point DPP-728 was discovered (3); only 3 years later DPP-728 provided the first proof of concept that a DPP-4 inhibitor improves glycemia in patients with T2DM (6). Kinetic studies of DPP-728 determined that it was a slow substrate for the catalytic site of the DPP-4 enzyme, rather than a simple competitive inhibitor, thereby blocking GLP-1 and GIP inactivation rather than simply slowing these rates of inactivation (3, 7).

Engineering the kinetic properties of DPP-728 led to the discovery of vildagliptin in 1998 (3, 8). Vildagliptin was first evaluated in man in 2002 (3, 9). Early clinical mechanism studies with vildagliptin demonstrated that by blocking GLP-1 and GIP inactivation the meal induced increases in GLP-1 and GIP were maintained over 24 h (1, 3, 10).

The purpose of this article is to integrate the expected as well as many unexpected GLP-1 and GIP actions emerging from the vildagliptin studies in man. A systematic review of the differences and similarities among DPP-4 inhibitors has been published previously (1). The therapeutic utility of DPP-4 inhibitors including vildagliptin is discussed in an accompanying article (11). The safety of vildagliptin has been reviewed previously (12); briefly the profile includes rare cases of mild to moderate elevations in hepatic enzymes, rare cases of angioedema (mostly in patients taking a concomitant ACE inhibitor) that resolved with ongoing treatment, and rare adverse drug reactions, including pancreatitis, bullous or exfoliative skin lesions, and arthralgia.

## β-cell

As expected vildagliptin increased the insulin secretion rate (corrected for the concentration of the glucose stimulus {abbreviated ISR/G}) in patients with T2DM (1). In contrast, 24-h insulin exposure was not increased with vildagliptin treatment indicating that insulin secretion was increased when needed without increasing total insulin exposure (13). There was a residual effect when GLP-1 action was blocked after 10 days of vildagliptin treatment suggesting that at least part of vildagliptin's effect on insulin secretion was due to GIP (14), which is consistent with the incretin hormone concept.

**Figure 1:**
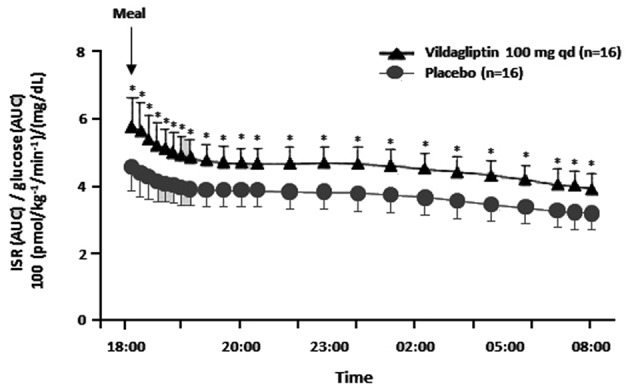
The insulin secretion rate (ISR) divided by the glucose area under the curve (AUC) (reflecting the relative glucose stimulus) from dinner until before breakfast in placebo patients vs. patients receiving an acute dose of vildagliptin before dinner. ^*^P < 0.05; qd, once daily; ISR/glucose AUC was calculated from data in Balas et al. (10).

During a study where vildagliptin was given acutely before an evening meal both GLP-1 and GIP levels rose at the beginning of the meal as expected, but unexpectedly the levels were maintained above placebo during the entire overnight period (1, 10). Furthermore, vildagliptin's acute effect to increase ISR/G was the same from early in the evening meal until breakfast the next morning (Figure 1), suggesting that enhanced ISR/G was not due to the pharmacological rise in GLP-1 at the beginning of the meal, but rather to the more physiological rise in GLP-1 maintained during the entire overnight period (1, 10). Although the GIP profile is similar to the GLP-1 profile, GIP is not predicted to be insulinotropic when given acutely (15).

Vildagliptin increased ISR/G in both impaired fasting glucose (IFG) (16) and impaired glucose tolerance (IGT) (17) but had no effect in healthy volunteers (18). These results are consistent with extensive studies with vildagliptin confirming the primary insulin secretion effect of vildagliptin was to improve the impaired sensitivity of the β-cells to glucose (1, 19, 20). Interestingly this improved sensitivity to glucose extends into the hypoglycemic range such that there was even less insulin secretion in the hypoglycemic range with vildagliptin than in the placebo group (21). Vildagliptin's effect to improve the sensitivity of the β-cells to glucose [ISR/G] was seen acutely and maintained over at least 2 years in patients with T2DM (22).

Interestingly HOMA-B was increased, but paradoxically this was due to the reduction in the fasting glucose levels and not to an increase in fasting insulin levels (23, 24). Vildagliptin's effect to increase insulin sensitivity to glucose was seen with glucose given orally or intravenously (25); this lack of the classic incretin effect was unexpected. This unexpected lack of the classic incretin effect has more recently been explained by the incretin effect being due to GIP and the GIP effect being overwhelmed by the GLP-1 effect secondary to DPP-4 inhibition (15). Thus, the vildagliptin effect to improve insulin secretion acutely was likely due to GLP-1 (15), but over days the GIP effect to stimulate insulin may have been restored (14). Vildagliptin was also associated with improved β-cell function that is likely secondary to the improved metabolic state such as improved proinsulin processing (a reduction in the proinsulin to insulin ratio) (24) and the acute insulin response to IV glucose after 6 weeks treatment with vildagliptin in subjects with IFG (16), and after 12 weeks treatment with vildagliptin in patients with T2DM (26).

T2DM has been associated with diminished maximum capacity of the β-cell to secrete insulin which has often been ascribed to a reduction in β-cell mass (27). The greatest promise of GLP-1 based therapies was that they would restore β-cell mass and thus the maximum capacity of the β-cell to secrete insulin. In neonatal rodents vildagliptin increased β-cell mass via increased β-cell neogenesis as well by a decrease in β-cell apoptosis (28). In a 1-year study with vildagliptin treatment in patients with T2DM that was followed by a three-month washout and where the maximum insulin secretory rate was assessed it was clear that vildagliptin had no disease modifying effect to increase β-cell mass (27). It was later demonstrated in mature rodents that there was also no disease modifying effect of vildagliptin to increase β-cell mass (28) suggesting that GLP-1 based therapy is only effective to increase β-cell mass in developing β-cells. Although there was no evidence of restoration of β-cell mass, the preponderance of the vildagliptin data does indicate that for at least 2 years β-cell function was maintained in vildagliptin treated patients but not in the untreated patients (20, 22, 27).

## α-cell

As expected vildagliptin suppressed an inappropriate glucagon response to an oral glucose challenge in patients with T2DM (29) and to a mixed meal challenge in patients with T2DM (9, 10, 23), but unexpectedly to a mixed meal challenge in subjects with IGT (17) and IFG (16). The improved glucagon response was maintained for at least 2 years in patients with T2DM (30). Importantly, vildagliptin had no effect on the glucagon response in normoglycemic individuals (18). Since GLP-1 levels were elevated in all cases, it appears that the glucagon response to GLP-1 was glucose dependent (1). The lack of adequate GLP-1 receptors on the α-cells suggested that the GLP-1 α-cell response was secondary to a paracrine effect by insulin. However, vildagliptin suppressed an inappropriate glucagon response to a mixed meal challenge in patients with type 1 diabetes mellitus (31, 32) indicating that this effect is not secondary to a paracrine effect by insulin; there is evidence that it may be mediated by a local paracrine effect of somatostatin (33).

## Hypoglycemia

Vildagliptin treatment increased insulin secretion and decreased glucagon secretion in hyperglycemia, providing a clear explanation for the improvement in glucose tolerance that persist for at least 2 years that has been discussed above. An important added benefit to this mechanism is that as the patients are treated to the normal glycemic range, they do so without increasing hypoglycemia (1, 3). This is presumably due to the vildagliptin mechanism leading to improved sensitivity of the β and α-cells to glucose. As discussed previously the insulin secretion response to glucose was attenuated in the hypoglycemic range (21) and it was expected that the decrease in glucagon due to GLP-1 seen in hyperglycemia would disappear. Unexpectedly, in T2DM patents with mild hyperglycemia the α-cell response to hypoglycemia was enhanced (21). However, the α-cell response to hypoglycemia was not enhanced in insulin-treated, metformin-treated and in type 1 diabetes and thus the α-cell response to hypoglycemia may be unaltered or enhanced depending on the study population (34).

**Figure 2:**
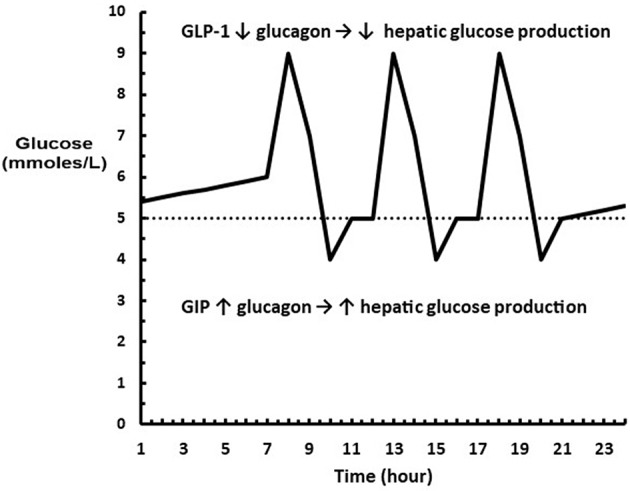
Diagram showing where GLP-1 and GIP are acting on glucagon relative to a glucose profile. (^…..^) represents euglycemia where neither GLP-1 or GIP are acting on the α-cell. The excursions above euglycemia cause GLP-1 to decrease glucagon levels leading to a decrease in hepatic glucose production. The excursions below euglycemia cause GIP to increase glucagon levels leading to an increase hepatic glucose production.

Interestingly, GIP is known to stimulate glucagon secretion in hypoglycemia and not influence glucagon secretion in hyperglycemia (35). The dual role of GLP-1 and GIP is visualized in Figure 2. Thus, the physiological consequences of an increase in the sensitivity of the β and α-cells to glucose following a carbohydrate rich meal such as fruit is that hepatic glucose production is rapidly inhibited while glucose utilization is enhanced so that glucose excursions are minimized with the least amount of insulin; and following a low or no carbohydrate meal such as meat that hepatic glucose production is increased to maintain euglycemia in order to compensate for the increased insulin required to store fat and protein.

## Lipo-toxicity

**Figure 3:**
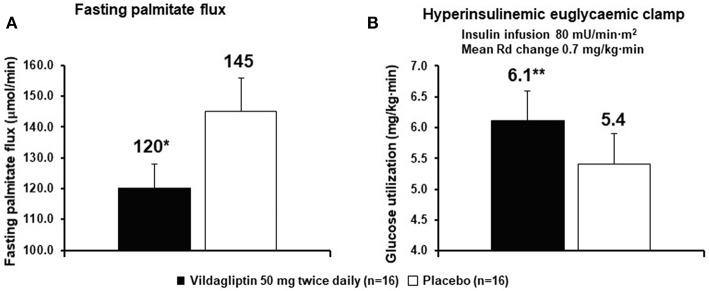
Vildagliptin decreases fasting fatty acid (palmitate dilution) flux from adipocytes **(A)** and increases glucose utilization **(B)**. The rates of glucose utilization are calculated from the last 30 min a long duration clamp (the second step of a two-step clamp) at a very high insulin concentration so the capacity of cells to oxidize glucose and capacity of the muscle to store glycogen are exceeded. Under such conditions the reduction of glucose to fatty acids by the liver becomes the rate limiting step (1, 3, 7). ^*^P < 0.01; ^**^P < 0.05; bid, twice daily; Rd, rate of glucose utilization. Data extracted from Azuma et al. (23).

**Figure 4:**
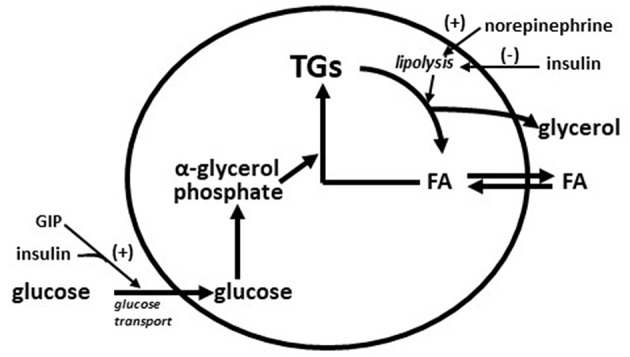
Diagram of storage and mobilization of fatty acids (FA) from adipocytes. GIP enhances insulin stimulated glucose transport (37) leading to increased α-glycerol phosphate which in turn increases rate of esterification of fatty acids into triglyceride (TG). Norepinephrine from nerve ending in adipose tissue stimulates lipolysis (breakdown of triglycerides to fatty acids and glycerol) and insulin inhibits lipolysis. Increased adipose cells size increases the lipolysis rate (38). A GIP effect on glucose transport during fasting is predicted to increase the rate of esterification of fatty acids into triglyceride to compensate for the increased rate of lipolysis from larger adipose cells.

Very early in the vildagliptin clinical program it was surprising that there was a decrease in fasting palmitate flux (Figure 3A) without any change in fasting insulin levels or fasting glucagon levels (23). This suggested an extra-pancreatic effect on the fat cell to either inhibit lipolysis or to stimulate esterification of FA. Rodent studies have shown that GIP improves the sensitivity of the adipocyte to insulin leading to increased esterification of FA relative to lipolysis resulting in reduced FFA release (36). The rate limiting step in esterification of fatty acids is glucose transport and the sensitivity to insulin of glucose transport was also increased by GIP in rodents (37). Thus, extending the physiological surge in GIP beyond the beginning of the evening meal into the fasting overnight period with vildagliptin presumably leads to reduced fatty acid flux from the adipocyte. This mechanism can be visualized in Figure 4. This could correct the underlying increased abdominal adipose cell size risk of developing T2DM where larger fat cells are associated with increased lipolysis and circulating FFA levels (38). This finding begs the question of the physiological relevance of GIP increasing esterification of FA. Under physiological conditions GIP is only increased at the beginning of meals. It is known that protein can markedly stimulate GIP release (39). As discussed above, when eating only meat, GIP presumably allows the insulin levels to rise enough to store the fat and protein and the glucagon levels to rise enough to prevent hypoglycemia. By increasing glucose transport in adipocytes in the presence of the low insulin levels associated with low carbohydrate meals, GIP may play an important role to further enhance fat storage.

**Figure 5:**
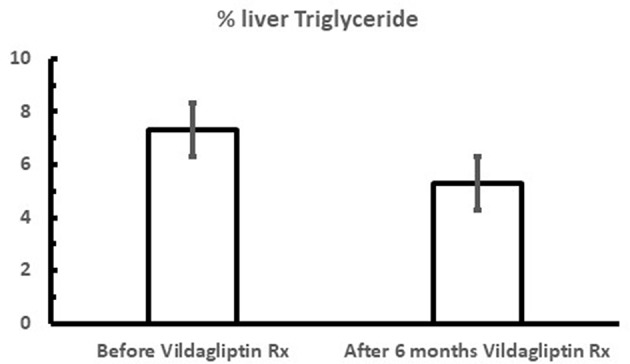
Mean fasting % liver triglyceride content during vildagliptin therapy (the Redirect Study) where metformin treated patients were additionally treated with and without 50 mg bid vildagliptin for 6 months. *P* < 0.001; Data extracted from Macauley et al. (44).

**Figure 6:**
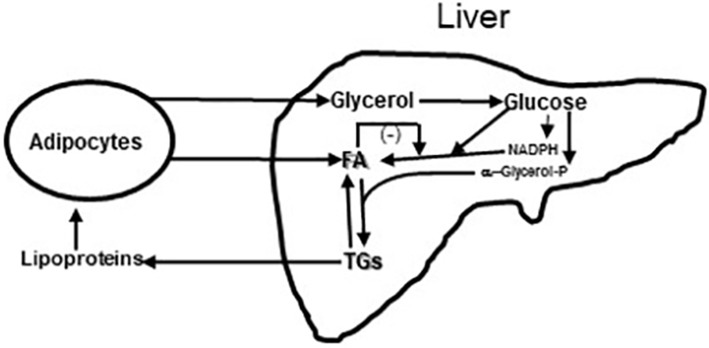
Diagram of Glycerol and Fatty acids (FA) coming to liver from fat cells and lipoproteins coming from liver to fat cells. In liver glycerol is metabolized to glucose. Some of the glucose is metabolized to provide reducing equivalents in the form of NADPH so that a second part of the glucose can undergo reduction to fatty acids (FA); this process of *de novo* fatty acid synthesis can be restrained by fatty acid feedback inhibition (44). A third part of the glucose is metabolized to α-glycerol phosphate to esterify fatty acids to triglyceride (TG). Triglycerides can be exported from the liver to fat and muscle or undergo lipolysis in the liver. Increased lipolysis in large adipocytes increases the flux of fatty acids and glycerol to the liver leading increased liver fatty acid and triglyceride levels. Under these conditions the capacity of the liver to metabolize glucose to fat is impaired and fasting glucose levels rise. This figure is a simplification of Figure 3 from Rui (46).

It was also shown that after only 6 weeks vildagliptin treatment was associated with improved glucose disposal (Figure 3B) under conditions where the rate limiting step exceeded the capacity of the muscle to store glycogen and thus is presumed to be lipogenesis in the liver (1, 3, 7, 23). Thiazolidinediones are known to reduce fasting fatty acid flux from adipocytes (40) leading to a reduction in liver fat (41, 42) and improved glucose utilization under conditions like those shown with vildagliptin (43). Vildagliptin was also shown to decrease liver fat [Figure 5, (44)]. As can be seen in Figure 6 a reduction in FA flux to the liver is predicted to decrease liver fat in the absence of increased hepatic export of lipid. Hepatic export of lipid is unlikely to have increased given the 13% fall in plasma triglyceride concentration (44). Furthermore, there was no vildagliptin treatment effect on postprandial triglyceride levels secondary to reduced flux of lipoproteins from the liver (45). Lipogenesis is diminished by increased liver fat via FA feedback inhibition and reducing liver fat is predicted to reduce FA feedback inhibition of lipogenesis (Figure 6). Since there was no decrease in fasting hepatic glucose production and a decrease in fasting glucose levels (44) it follows that fasting glucose utilization increased and that this increase was mostly likely due to increased lipogenesis. This is important since at the end of each day any glucose not oxidized or stored as glycogen must be converted to fat in the liver to avoid a rise in fasting glucose levels. This is also consistent with the observation that HOMA-B increased due to a reduction fasting glucose levels with no change in the fasting insulin levels that was discussed earlier.

## Weight

Overall vildagliptin was associated with weight neutrality. The low probability of hypoglycemia which precludes the weight gain associated with defensive eating to avoid hypoglycemia is consistent with such weight neutrally. However, the caloric penalty associated with a reduction of glucose levels from above to below the renal threshold predicts weight gain. When the weight changes from pooled monotherapy studies after 24 weeks of therapy with vildagliptin were assessed vs. the fasting plasma glucose (FPG) levels at baseline it was clear that no weight change was observed at the renal threshold in T2DM (FPG of 14.6 mmol/L or 263 mg/dL). Baseline FPG values below and above this threshold were associated with weight loss and weight gain, respectively; a baseline FPG of 8 mmol/L (144 mg/dL) predicted a weight loss of 1 kg (47). There was no evidence of a satiety effect with vildagliptin that could explain this apparent weight loss when glucosuria was considered (48). However, two other potential weight mitigating mechanisms were identified in the vildagliptin studies. In the first there was a reduction in Apo B-48 secretion which presumably decreases fat extraction from the gut (45). In the second there was an increase postprandial lipolysis from adipose tissue and increased postprandial fat oxidation in the muscle so that during the fed state fat acids are mobilized and burned (49). Although such a mechanism is predicted to cause glucose intolerance, the pancreatic mechanisms to improve glucose tolerance clearly prevails. Thus, as discussed above, in the fasted state vildagliptin treatment results in greater fat storage while in the fed state it mobilizes and burns fat. This has the effect of reducing lipo-toxicity without the weight gain associated with thiazolidinediones (50).

## Conclusions

The clinical studies with vildagliptin in T2DM have shown that DPP-4 inhibition with vildagliptin attenuated the diminished sensitivity of the islets to glucose, reduced the insulin resistance due to inappropriate glucagon secretion and lipo-toxicity characterized by greater triacylglycerol storage in non-fat tissues, decreased the hyperglycemia that is associated with glucose toxicity, and maintained but did not restore the diminished maximum capacity of the β-cells to secrete insulin. The improved sensitivity of the islets to glucose not only led to a reduction in hyperglycemia but did so without increased hypoglycemia. The VERIFY study just reported that an early metformin and vildagliptin combination was more durable than adding vildagliptin to patients failing metformin and that over 5 years vildagliptin plus metformin resulted in a small weight loss in patients that were clearly below the renal threshold during this 5-year period (51). The contributions of reduced lipo-toxicity and improved islet sensitivity to glucose in the face of no weight gain to vildagliptin's durability remains to be determined.

## Author Contributions

The author confirms being the sole contributor of this work and has approved it for publication.

### Conflict of Interest

Prior to Nov 2017, the author was an employee of Novartis Pharmaceuticals. The author declares that this review was written in the absence of any commercial or financial relationships that could be construed as a potential conflict of interest.
